# The complete mitochondrial genome of *Sicista caudata* Thomas, 1907 (Rodentia: Sicistidae) and its phylogenetic analyses

**DOI:** 10.1080/23802359.2024.2432349

**Published:** 2024-11-23

**Authors:** Zhu Liu, Fu-Ying Zhang, Zhi-Hui Zhang, Qiu-Ying Guo

**Affiliations:** College of Life Science and Technology, Mudanjiang Normal University, Mudanjiang, PR China

**Keywords:** *Sicista caudata*, mitogenome, phylogenetic trees

## Abstract

This study aimed to examine the complete mitogenome sequence of *Sicista caudata* Thomas, 1907. A circular double-stranded structure made up the mitochondrial genome of *S. caudata*. The complete length of the mitochondrial genome was 16,629 bp. The mitochondrial genome of *S. caudata* included 13 protein-coding genes, one control region, 22 tRNA genes, two rRNA genes, and one origin of L-strand replication. *S. caudata* was supported by bootstrap values of 100%. This study verified the evolutionary status of *S. caudata* in the Superfamily Dipodoidea at the molecular level.

## Introduction

*Sicista caudata* Thomas, 1907 belongs to the order Rodentia, Superfamily Dipodoidea, family Sicistidae, subfamily Sicistinae, genus *Sicista* (Wilson and Reeder 2005)*. External morphological characteristics*: Small body size (60–71 mm), with an extremely long tail (the ratio of tail length to body length is 152–178%). The dorsal fur is tan-yellow, with the mid-dorsal fur being darker and without longitudinal stripes. The ventral side is grayish-yellow, with the base of the fur being gray (Gao et al. 2024). It has a propensity for social living, uses tree cavities as a concealed habitat, is skilled at climbing, and is primarily nocturnal (Piao 2019). The population density of Sicistidae in the wild is low. Specimens of Sicistidae are difficult to obtain. This is especially true of *S. caudata*. Since *S. caudata* was discovered on the Russian island of Sakhalin in 1907, no more than 10 specimens have been reported so far. International Union for Conservation of Nature (IUCN) lists it as ‘data deficient’. Due to the rarity of Sicistidae animals, no mitochondrial genome of *S. caudata* has been reported. In this study, the complete mitochondrial genome of *S. caudata* was sequenced, and the phylogenetic relationships within the Superfamily Dipodoidea were analyzed. The mitochondrial genome of *S. caudata* is the first reported in Sicistidae.

## Materials and methods

**Figure 1.**
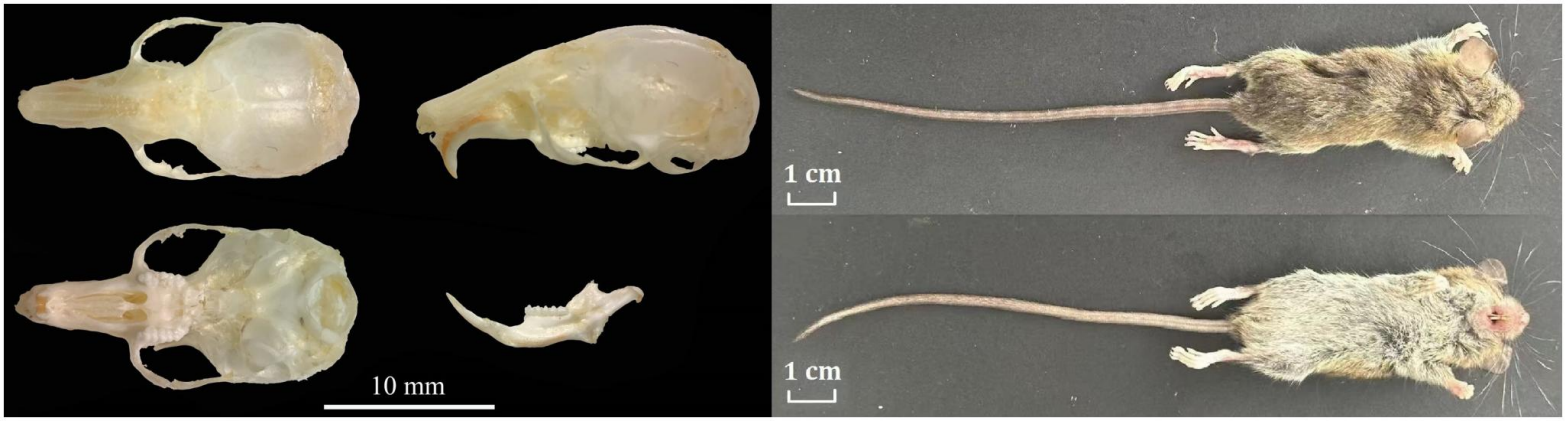
Pictures of external and skull morphologies. Pictures courtesy of Zhu Liu.

A male *S. caudata* sample was collected from Hengdaohezi City (44°48′44″N, 129°02′04″E), Heilongjiang Province, China, in August 2023 (Figure 1). The *Cyt b* gene sequence of the specimen was blasted against the GenBank database. The sequence OR672123 was the most similar to the specimen from *S. caudata* (MK259965), and the percent identity was 96.32%. The sample was stored at −75 °C before use and deposited at the Animal and Plant Herbarium of Mudanjiang Normal University (Liu Zhu, swxlz0@126.com) under the voucher number CWJS202304. Material in the CWJS202304 collection includes: skull, flayed specimen, muscle tissue, and internal organs (heart, liver, spleen, lungs, and kidneys). Genomic DNA was extracted from leg muscle using the EasyPure genomic DNA kit (TransGen Biotech Co., Beijing, China). We designed 15 pairs of primers for polymerase chain reaction (PCR) based on the reported mitochondrial genome of Dipodoidea (Figure S1, Table S1). PCR reaction conditions: initial denaturation at 94 °C for 4 min, denaturation at 94 °C for 45 s, annealing at 52–56 °C for 1 min, extension at 72 °C for 90 s, and final extension at 72 °C for 7 min, with 35 cycles. The first-generation sequencing technology was used for sequencing in this study (ABI 3730 sequencer; Boshi Biotechnology Co. Ltd., Harbin, China). The sequences were assembled using DNAstar, analyzed, and adjusted manually. The annotation of the *S. caudata* mitochondrial genome was performed using web-based services MITOS (Bernt et al. 2013) and software PhyloSuite v 1.2.2 (Zhang et al. 2020). The circular mitochondrial genome map of *S. caudata* was drawn using SnapGene 6.0.2 (Tello et al. 2022). In this study, the molecular phylogeny of *S. caudata* was investigated using the complete mitochondrial genomes of 11 species from nine genera (*Sicista*, *Allactaga*, *Dipus*, *Euchoreutes*, *Jaculus*, *Scarturus*, *Stylodipus*, *Eozapus*, and *Orientallactaga*) in Superfamily Dipodoidea deposited in the GenBank. The phylogenetic tree was constructed using 13 protein-coding genes of the complete mitochondrial genome through MEGA 11.0 software (Tamura et al. 2021). The phylogenetic tree was constructed using the General Time Reversible model of maximum-likelihood method with 1000 bootstrap replications.

## Results

**Figure 2.**
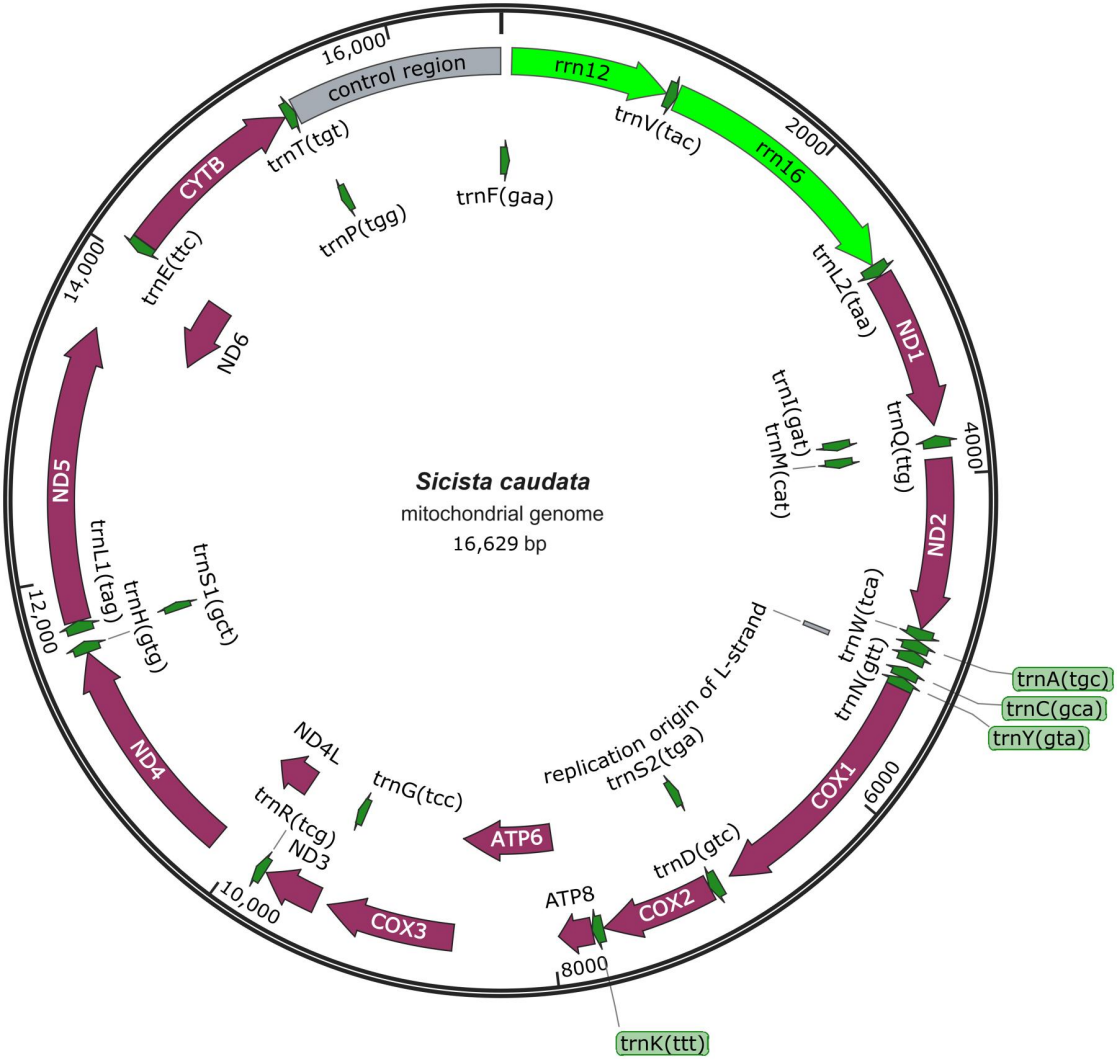
Circular mitochondrial genome map of *S. caudata.* Clockwise represents its position in the H chain, counterclockwise means it is in the L chain. Green, rRNA; violet, protein-coding gene; gray, replication origin of L-strand and control region.

**Figure 3.**
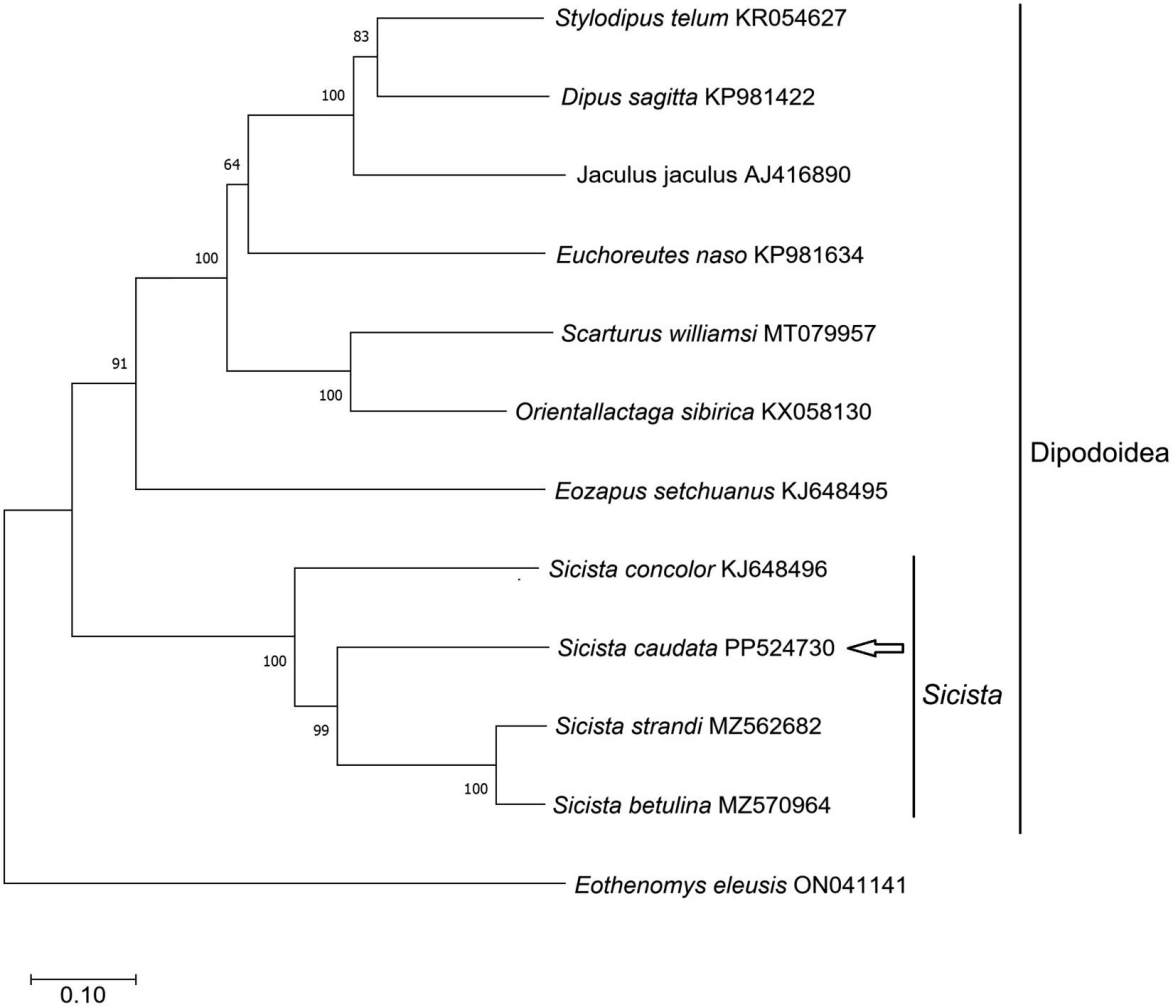
Phylogenetic tree was constructed using 13 protein-coding genes of the complete mitochondrial genome through MEGA 11.0 software, and also constructed using the General Time Reversible model of maximum likelihood method with 1000 bootstrap replications. *Sicista caudata* (PP524730) is annotated and uploaded by this study. The following sequences were used: KP981422 (Luo and Liao 2016), KP981634 (Luo and Liao 2016), AJ416890 (unpublished), MT079957 (İbiş 2020), KR054628 (Luo et al. 2016), KJ648496 (Yue et al. 2015), MZ562682 (Andersen et al. 2022), MZ570964 (Andersen et al. 2022), KJ648495 (Yue et al. 2015), and KX058130 (Ding et al. 2016). The outgroup was ON041141 (Luo and Liao 2016).

A circular double-stranded structure made up the mitochondrial genome of *S. caudata* (Figure 2). The length of the complete mitochondrial genome was 16,629 bp. The mitochondrial genome of *S. caudata* included 13 protein-coding genes, one control region, 22 tRNA genes, two rRNA genes, and one origin of L-strand replication. The total base composition of *S. caudata* mitochondrial genome was A (29.7%), T (28.1%), G (14.0%), and C (28.2%). We found significant A–T skew in base composition, especially in control regions and protein-coding genes. The *ND6* gene and eight tRNA genes of *S. caudata* were encoded on the L strand. The other mitochondrial genes were encoded on the H strand. GenBank received the annotated mitochondrial genome sequences with accession number PP524730. The control region of the mitochondrial genome existed between the tRNA-Pro and tRNA-Phe. The control region had no structural genes but had only promoters and regulatory sequences for replication and transcription. The total length of 13 protein-coding gene sequences was 11,386 bp. The lengths of 22 tRNA genes were between 60 and 75 bp. The length of L-strand replication origin (*OL*) was 36 bp. The phylogenetic tree (Figure 3) illustrated the evolutionary relationships among various species. *S. caudata* (PP524730) was nested within the genus *Sicista* and exhibited a close phylogenetic affinity to *S. strandi* (MZ562682) and *S. betulina* (MZ570964). Additionally, the tree indicated that the genus *Sicista* was a member of the larger clade Dipodoidea. Our results indicated that *S. caudata* and other species from the Superfamily Dipodoidea had further phylogenetic relationships.

## Discussion and conclusions

The arrangement of genes in *S. caudata* mitochondrial genome was consistent with that in other Superfamily Dipodoidea species (Yue et al. 2015; Ding et al. 2016; Luo et al. 2016; Luo and Liao 2016; İbiş 2020; Andersen et al. 2022). *S. caudata* exhibited a close phylogenetic affinity to *S. strandi* and *S. betulina. S. caudata* and the other species from Superfamily Dipodoidea had a further phylogenetic relationship. This study verified the evolutionary status of *S. caudata* in the Superfamily Dipodoidea at the molecular level. The mitochondrial genome would be a significant supplement for the genetic background of *S. caudata*.

## Supplementary Material

supplementary materials.docx

## Data Availability

The data supporting the findings of this study are publicly accessible on the GenBank website at https://www.ncbi.nlm.nih.gov/, under the reference number PP524730. The additional data can be accessed at BioSample: SAMN41038315, SRA: SRR29259733, and BioProject: PRJNA1102988. Raw data can be downloaded at https://trace.ncbi.nlm.nih.gov/Traces/?view=run_browser&acc=SRR29259733&display=metadata.

## References

[CIT0001] Andersen LW, Jacobsen MW, Frydenberg J, Møller JD, Jensen TS. 2022. Phylogeography using mitogenomes: a rare Dipodidae, *Sicista betulina*, in North‐western Europe. Ecol Evol. 12(4):e8865. doi:10.1002/ece3.8865. 35475180 PMC9022092

[CIT0002] Bernt M, Donath A, Jühling F, Externbrink F, Florentz C, Fritzsch G, Pütz J, Middendorf M, Stadler PF. 2013. MITOS: improved de novo metazoan mitochondrial genome annotation. Mol Phylogenet Evol. 69(2):313–319. doi:10.1016/j.ympev.2012.08.023. 22982435

[CIT0003] Ding L, Luo G, Li W, Liao J. 2016. Characterization and phylogenetic analysis of the complete mitogenome of *Allactaga sibirica* (Rodentia: Dipodidae). Biochem Syst Ecol. 69:195–203. doi:10.1016/j.bse.2016.10.004.

[CIT0004] Gao Y, Wu Y, Han M, Guo Q, Cai H, Zhang C, Jin Z, Chen H, Zhang J, Liu Z. 2024. Preliminary morphological and molecular systematics analysis of *Sicista caudata*. Acta Theriol Sin. 44(4):515–522.

[CIT0005] İbiş O. 2020. Whole mitochondrial genome sequence and phylogenetic relationships of Williams’s jerboa (*Scarturus williamsi*) from Turkey. PeerJ. 8:e9569. doi:10.7717/peerj.9569. 32742814 PMC7369027

[CIT0007] Luo G, Ding L, Liao J. 2016. The complete mitochondrial genome of *Stylodipus telum* (Rodentia: Dipodidae) and its phylogenetic analysis. Mitochondrial DNA A DNA Mapp Seq Anal. 27(4):2568–2569. doi:10.3109/19401736.2015.1038807. 26024139

[CIT0008] Luo G, Liao J. 2016. Phylogenetic analysis of *Dipus sagitta* and *Euchoreutes naso* (Rodentia: Dipodidae) based on the mitochondrial genomes. Mitochondrial DNA A DNA Mapp Seq Anal. 27(4):2648–2650. doi:10.3109/19401736.2015.1043529. 26029878

[CIT0009] Piao Z. 2019. The gentle *Sicista caudata*. Forest Humankind. 5:94–99.

[CIT0010] Tamura K, Stecher G, Kumar S. 2021. MEGA11: Molecular Evolutionary Genetics Analysis Version 11. Mol Biol Evol. 38(7):3022–3027. doi:10.1093/molbev/msab120. 33892491 PMC8233496

[CIT0011] Tello M, Oporto B, Lavín JL, Ocejo M, Hurtado A. 2022. Characterization of a carbapenem-resistant *Escherichia coli* from dairy cattle harbouring bla NDM-1 in an IncC plasmid. J Antimicrob Chemother. 77(3):843–845. doi:10.1093/jac/dkab455. 34907439 PMC8865002

[CIT0012] Wilson DE, Reeder DM. 2005. Mammal species of the world. 3rd ed. Baltimore: The Johns Hopkins University Press.

[CIT0013] Yue H, Yan C, Tu F, Yang C, Ma W, Fan Z, Song Z, Owens J, Liu S, Zhang X. 2015. Two novel mitogenomes of Dipodidae species and phylogeny of Rodentia inferred from the complete mitogenomes. Biochem Syst Ecol. 60:123–130. doi:10.1016/j.bse.2015.04.013.

[CIT0014] Zhang D, Gao F, Jakovlić I, Zou H, Zhang J, Li WX, Wang GT. 2020. PhyloSuite: an integrated and scalable desktop platform for streamlined molecular sequence data management and evolutionary phylogenetics studies. Mol Ecol Resour. 20(1):348–355. doi:10.1111/1755-0998.13096. 31599058

