# Supplementary material for: The complete mitochondrial genome of Sicista caudata Thomas, 1907 (Rodentia: Sicistidae) and its phylogenetic analyses
Source: Mitochondrial DNA B Resour. 2024 Nov 23;9(11):1611–4. doi: 10.1080/23802359.2024.2432349 (PMC11587713; doi:10.1080/23802359.2024.2432349)
Supplement: supplementary materials.docx [file TMDN_A_2432349_SM8037.docx]

Supplementary Materials


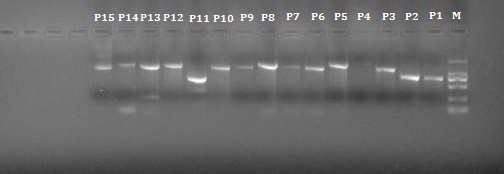


Figure S1. PCR gel images of 15 pairs of primers. We designed 15 pairs of primers for PCR of the complete mitochondrial genome of *Sicista caudata* based on the reported mitochondrial genome of Superfamily Dipodoidea*.* P1–P15 stands for primer 1 to primer 15. M stands for marker (DL2000).


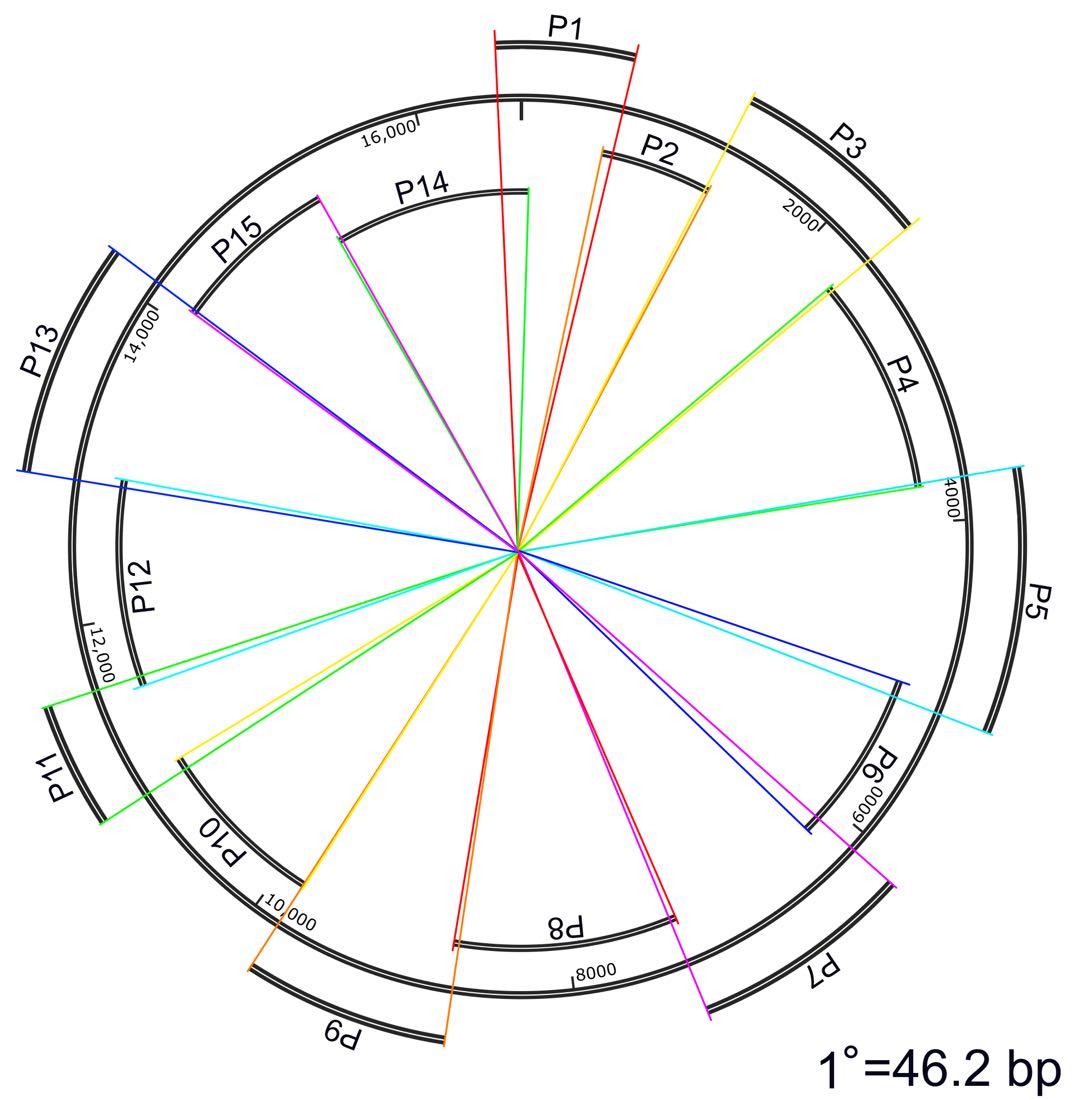


Figure S2. Mitochondrial genome sequence assembly map. We assembled the sequenced 15 sequences as shown in Figure.

Table S1. Sequences of all primers designed.

| Primer | drection | Sequence | TM(℃) | genes included |
| --- | --- | --- | --- | --- |
| CWJS1-1 | forward | CTTAGACCATCATTGCAGAAC | 55.7 | CR, trnF, rrn12, trnV, rrn16 |
| CWJS1-2 | reverse | CAAGTCCTTTGAGTTTTAAGCG | 57.2 |  |
| CWJS1-3 | forward | AGATTAGATACCCCACTATGC | 54.8 | rrn12, trnV, rrn16 |
| CWJS1-4 | reverse | TCTGGCTAGCTCATTATGCAA | 57.3 |  |
| CWJS2-1 | forward | TACCGCAAGGGAAAGATGAAA | 59.3 | rrn16 |
| CWJS2-2 | reverse | TAGGGTAACTTGGTCCGTTGA | 60.6 |  |
| CWJS3-1 | forward | AATTTCGGTTGGGGTGACCTC | 63.1 | rrn16, trnL2, ND1 |
| CWJS3-2 | reverse | CTCTATCAAAGTAACTCTTTTGTC | 53.8 |  |
| CWJS4-1 | forward | AGCCCTATGTATATGACACGT | 56.3 | ND1, trnI, trnQ, trnM, ND2, trnW, trnA, trnN |
| CWJS4-2 | reverse | GAGAAGTAGATTGAAGCCAGT | 56.2 |  |
| CWJS5-1 | forward | AGACCAAGGACCTTCAAAGTC | 59.8 | trnW, trnA, trnN, trnC, trnY, COX1 |
| CWJS5-2 | reverse | TGATGGGCTCAGACAATGAAG | 59.4 |  |
| CWJS6-7-1 | forward | GTTCTGATTTTTTGGTCACCC | 57.8 | COX1, trnS2, trnD, COX2 |
| CWJS6-7-2 | reverse | TCATAACTTCAGTATCATTGATG | 52.1 |  |
| CWJS8-1 | forward | CCGCAGTCACTTTAATTCTCA | 57.5 | COX2, trnK, ATP8, ATP6, COX3 |
| CWJS8-2 | reverse | TCATATCACTAGTCCGGATGT | 56.6 |  |
| CWJS9-1 | forward | GATAACACATAATGACMCACCA (M=C or A) | 49.2 ~ 51.1 | COX3, trnG, ND3, trnR |
| CWJS9-2 | reverse | CATAATCTAATGAGTCGAAATCA | 52.5 |  |
| CWJS10-1 | forward | CAAAAAGGACTAGAATGAACAGA | 55.4 | trnR, ND4L, ND4 |
| CWJS10-2 | reverse | GACTAGGGCTATRTGGCTTAC (R=G or A) | 52.4 ~ 54.4 |  |
| CWJS11-1 | forward | TGTCCACACACATAGCCTAC | 55.6 | ND4, trnH, trnS1 |
| CWJS11-2 | reverse | GTTAGCAGTTCTCGCTTTCT | 54.2 |  |
| CWJS12-1 | forward | CCAACCCCAAACTCATTACAGG | 61.2 | trnH, trnS1, trnL1, ND5 |
| CWJS12-2 | reverse | GTGACTATTAGGGCTCAGGCG | 61.7 |  |
| CWJS13-1 | forward | ACCACAAGCGCCCTAACAAT | 58.5 | ND5, ND6, trnE, CYTB |
| CWJS13-2 | reverse | GTCAATGAATGAGTCGTTGAC | 56.8 |  |
| CWJS14-1 | forward | CTCATCTTATTACCACTAGCC | 53.8 | CYTB, trnT, trnP, CR, trnF |
| CWJS14-2 | reverse | ACTAATAAGAAGGCTAGGACC | 55.1 |  |
| L | forward | GGACTTATGACATGAAAAATCATCGTTG | 59.2 | CYTB |
| H | reverse | GATTCCCCATTTCTGGTTTACAAGAC | 61.1 |  |
